# Supplementary material for: Identification and Analysis of the CBF Gene Family in Three Species of Acer under Cold Stress
Source: Int J Mol Sci. 2023 Jan 20;24(3):2088. doi: 10.3390/ijms24032088 (PMC9916880; doi:10.3390/ijms24032088)
Supplement: Supplementary file 1 [file ijms-24-02088-s001.zip › ijms-2079461-supplementary.pdf]

Supplementary Material

Supplementary Table 1： The sequences of primers used for qRT-PCR

Gene name	Forward sequence (5'to 3')	Reverse sequence (5'to 3')
Apse002T0243400.1	GTTACTCTGACCCACAACCG	AGCCAACATCAATTCCTCGTC
Apse002T0243400.1	GTTACTCTGACCCACAACCG	AGCCAACATCAATTCCTCGTC
Apse002T0243300.1	AATGTGGAGGTGGCTTCTG	ATCCCAAATACCGCCTCTTC
Apse002T0243300.1	AATGTGGAGGTGGCTTCTG	ATCCCAAATACCGCCTCTTC
Apse011T0099600.1	GACAACTATCGGACCCTCTTTC	CTGAATGAGAAGCTCTACTGGC
Apse011T0099600.1	GACAACTATCGGACCCTCTTTC	CTGAATGAGAAGCTCTACTGGC
Apse002T0243600.1	CAGTCATTGCCAAGTCCAAAC	ATGAAAAGTCGGGTGCCTC
Apse002T0243600.1	CAGTCATTGCCAAGTCCAAAC	ATGAAAAGTCGGGTGCCTC
Apse007T0094500.1	TCAACCAAAAGTACCTCCACC	CAGAAACCCATTTGCCACTAC
Apse007T0094500.1	TCAACCAAAAGTACCTCCACC	CAGAAACCCATTTGCCACTAC
Acyan11G0087800.1	GCAGTGGGACAGTTGAATTTG	CTGACATGAACGACAGAGGG
Acyan11G0087800.1	GCAGTGGGACAGTTGAATTTG	CTGACATGAACGACAGAGGG
Acyan11G0087900.1	GTGTTTGACATGCCTGGATTG	TTCTCTACTGCTTCCACTTGC
Acyan11G0087900.1	GTGTTTGACATGCCTGGATTG	TTCTCTACTGCTTCCACTTGC
Acyan02G0289000.1	CCCATTGCCAAGTCCAAAC	TCCATGATAAATCGGGTGCC
Acyan02G0289000.1	CCCATTGCCAAGTCCAAAC	TCCATGATAAATCGGGTGCC
Acyan07G0093600.1	TCAACCAAAAGTACCTCCACC	GCCTAACACCCCGATAAGATG

Acyan07G0093600.1	TCAACCAAAAGTACCTCCACC	GCCTAACACCCCGATAAGATG
Atru.chr10.1810	CTAACTTTCCTTTGCCTTGCG	ATTTAGATCCAACGGCGAGG
Atru.chr10.1810	CTAACTTTCCTTTGCCTTGCG	ATTTAGATCCAACGGCGAGG
Atru.chr1.1042	TGCTAGGAAATTGTACGGACC	AAACCACGAGAAGGGAACATAG
Atru.chr1.1042	TGCTAGGAAATTGTACGGACC	AAACCACGAGAAGGGAACATAG
Atru.chr2.1230	CACTACAACTACAACCCCACC	CTCCCTGTTTTCCTCTGTTTTC
Atru.chr2.1230	CACTACAACTACAACCCCACC	CTCCCTGTTTTCCTCTGTTTTC
Atru.chr4.1701	GCAGTGAAGCTGTATAGAGGTG	GTGTCGAAAGTCCCTAACCAG
Atru.chr4.1701	GCAGTGAAGCTGTATAGAGGTG	GTGTCGAAAGTCCCTAACCAG
Atru.chr13.731	AAGTTGGTGCTAGGGTTGAC	AGGTAACCGGACAGAATTGG
Atru.chr13.731	AAGTTGGTGCTAGGGTTGAC	AGGTAACCGGACAGAATTGG
Atru.chr6.2697	CACCAATTTCACGCAACCTG	CCTTTGTCTCACACCTCTGTAG
Atru.chr6.2697	CACCAATTTCACGCAACCTG	CCTTTGTCTCACACCTCTGTAG
Atru.chr4.2790	CAGGGAGGTTTCTTGGAGTTC	CTCTTTCGTTGTAGGGTCTCTG
18S	GAGGTAGCTTCGGGCGCAACT	GCAGGTTAGCGAAATGCGATAC
